# Cone Beam CT in Diagnosis and Surgical Planning of Dentigerous Cyst

**DOI:** 10.1155/2017/7956041

**Published:** 2017-02-15

**Authors:** Naira Figueiredo Deana, Nilton Alves

**Affiliations:** ^1^Magister Program in Dentistry, Faculty of Dentistry, Universidad de La Frontera, Temuco, Chile; ^2^CIMA-Research Center in Applied Morphology, Universidad de La Frontera, Temuco, Chile; ^3^Faculty of Dentistry, Universidad de La Frontera, 1145 Francisco Salazar Avenue, P.O. Box 54-D, Temuco, Chile

## Abstract

Diagnosis and preoperative planning are critical in the execution of any surgical procedure. Panoramic radiography is a routine method used in dentistry to assist clinical diagnosis; however, with this technique 3D anatomical structures are compressed into 2D images, resulting in overlapping of structures which are of interest in the diagnosis. In this study we report the case of a patient who presented with a dentigerous cyst of expressive dimensions in the body of the mandible region. The surgery was planned and executed after observing the margins of the lesion by Cone Beam Computed Tomography (CBCT). We conclude that CBCT is a precise method to help diagnosis; it provides greater accuracy in surgical treatment planning through 3D image display, allowing more effective results.

## 1. Introduction

The dentigerous (or follicular) cyst (DC) is the second most common type of dental cyst and the most common in jaw development [1]. Benn and Altini [2] propose the existence of two types of dentigerous cysts: one of a developing nature and the other inflammatory. From a clinical point of view, dentigerous cysts are generally asymptomatic, slow-growing, associated with the crown of an impacted or unerupted permanent tooth, and characterised by retarded eruption of the tooth [1]. However they may grow large enough to cause destruction of the cortical bone, resulting in fluctuation, spontaneous pain, exudation, and rapid development of the pathology, which are signs of acute inflammation round the margins of the cyst [3].

Diagnosis of the lesion should not be based on X-ray evidence alone, but also on clinical evidence, particularly microscopic examination of the sample [2].

Periapical and panoramic X-rays are the most commonly used imaging examinations in dentistry for diagnosis and surgical planning. However the information acquired in these examinations is limited, since the three-dimensional anatomy of the area X-rayed is shown in two dimensions, with superimposed planes. Although these methods produce acceptable images in the mesiodistal direction, observation in the vestibulolingual direction is difficult. There may also be geometrical distortion of the structures X-rayed when we use these imaging methods [4]. A very efficient imaging technique for diagnosing DC is Magnetic Resonance Imaging (MRI), since it allows cysts to be distinguished from tumoral lesions [5], ensuring safe, efficient performance [6]; however MRI is a very high-cost examination with limited availability, making it impractical for routine clinical use.

In the last decade a new technology—Cone Beam Computerised Tomography (CBCT)—has offered dental surgeons three-dimensional reproduction of images of mineralised maxillofacial tissues, with minimum distortion and significantly lower doses of radiation than conventional CT [7].

The object of this study was to report a clinical case of a dentigerous cyst in which the diagnostic hypothesis and the decision on the treatment plan were based on CBCT.

## 2. Case Report

Male patient, 28 years, Brazilian, white, attended a private dental practice complaining of increased volume in the vestibular region of the left mandibular second molar and slight discomfort when eating. The patient reported that the increase had evolved slowly over approximately one year, with no apparent cause and no pain. In clinical examination, the absence of the left mandibular second molar was observed, with an increase in volume in the vestibular region.

**Figure 1:**
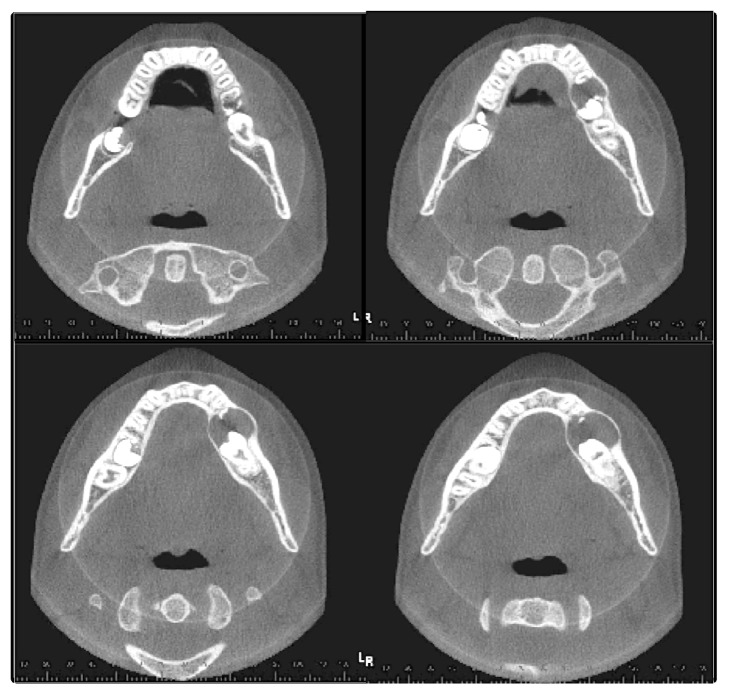
CBCT image, axial sections, showing bulging of osseous cortices in the region of the left permanent mandibular second molar.

**Figure 2:**
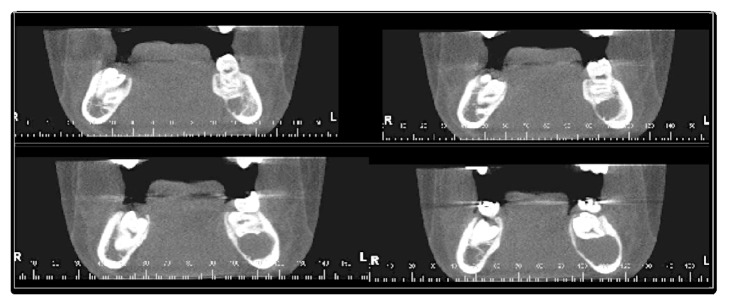
CBCT image, coronal sections, showing hypodense image associated with the left permanent mandibular second molar, with radicular reabsorption of the left permanent mandibular first molar.

**Figure 3:**
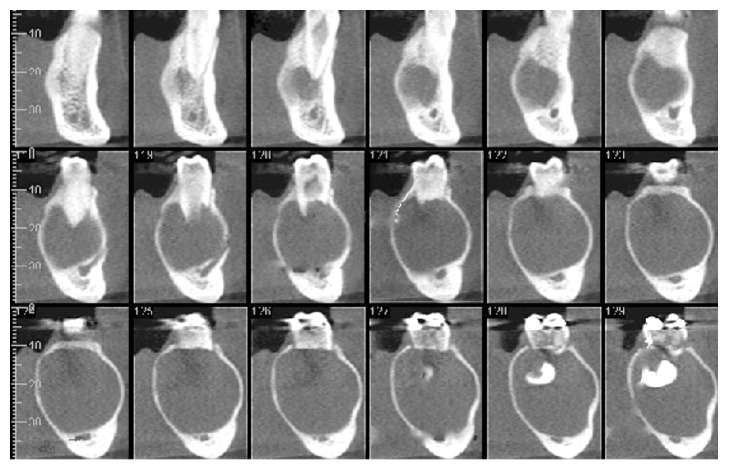
CBCT image: orthoradial reconstructions from the left mandibular first premolar to the left permanent mandibular first molar. An extensive hypodense area can be observed with radicular reabsorption of the left mandibular second premolar and the left permanent mandibular first molar.

**Figure 4:**
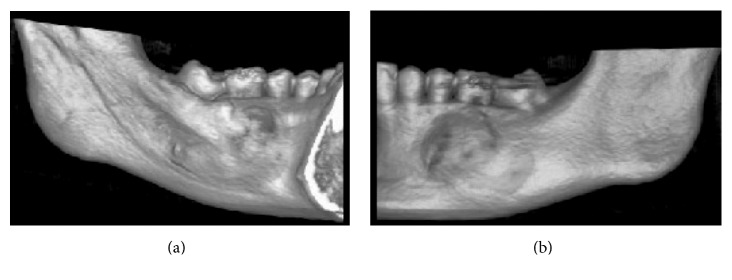
CBCT image, 3D reconstructions. Observe the body of the mandible in the region of the left permanent mandibular first molar. Internal (lingual) view (a) and external (vestibular) view (b).

CBCT was carried out to evaluate the case. The CBCT images were obtained with i-CAT® Next Generation, 120 Kv, 18.5 mA, FOV 16 × 6 cm. The data were converted into 3D images with volume representation by the Vision Standalone software. In the axial sections an extensive, hypodense image was observed in the body of the mandible region, left side, containing the permanent mandibular second molar; bulging of both osseous cortices was also found (Figure 1). In the coronal sections a hypodense image was observed in the body of the mandible region, left side, associated with the left permanent mandibular second molar, with radicular reabsorption of the left permanent mandibular first molar (Figure 2). In orthoradial reconstructions from the left mandibular first premolar to the left permanent mandibular first molar an extensive hypodense area was observed with radicular reabsorption of the left mandibular second premolar and the left permanent mandibular first molar (Figure 3). A 3D reconstruction was made of the vestibular and lingual views (Figure 4).

The diagnostic hypothesis based on the clinical and imaging examinations was of a dentigerous cyst of the circumferential radiographic type. Because the lesion presented with relatively unaggressive comportment, with large size and limits, and was located very close to important anatomical structures, we decided on conservative surgical treatment, that is, decompression of the cyst and subsequent enucleation.

The procedure practised on the patient started with aspiration puncture, which confirmed the presence of an intralesional liquid content. After confirmation of a cyst-type lesion, an incision was made to give access to the cyst capsule and to promote biopsy. The surgical piece obtained was sent for histopathological examination, resulting in the histological diagnosis of a dentigerous cyst.

## 3. Discussion

Dentigerous cysts account for 30.7% of all odontogenic cysts in the Brazilian population [8], being commoner in men than in women (2.33 : 1) [9], with no racial predilection, most frequent in the second and third decades of life [2, 10] due to the chronology of dental eruption [11]. They tend to be solitary; bilateral dentigerous cysts usually occur in association with syndromes like cleidocranial dysplasia and mucopolysaccharidosis (Type VI) [11].

Dentigerous cysts are usually located in the region of the maxillary canines and the mandibular third molars [2, 12, 13]; in the case reported the cyst was associated with the left mandibular second molar. In general the patients present with impacted teeth or slow-growing, asymptomatic swellings [11]. In the present case the patient reported slow, asymptomatic growth and also presented unerupted left mandibular second molar.

Hyomoto et al. [14] analysed the factors which interfere with the spontaneous eruption of mandibular premolars associated with this type of cyst and concluded that impacted teeth tend to erupt more easily in the presence of inflammatory infiltrate, suggesting that the higher the number of inflammatory cells, the greater the possibility of eruption of the tooth associated with a cyst. For Carvalho and Luna [15], this finding may explain the fact that the depth of the impaction is insufficient to determine the failure of the tooth to erupt spontaneously. Thus the characteristics of the impaction are of fundamental importance in enabling the dentist to select a suitable treatment plan [15].

CBCT has been widely used in dentistry to provide basic information on dental and maxillofacial structures for diagnosis and surgical planning [16]. According to Cha et al. [17] CBCT can be a much-needed improvement in diagnosis and treatment planning. However it must be noted that all tomographic examinations must be carried out under the basic principles of justification (weighing benefits against risks) and optimisation (keeping doses as low as possible) [18], because of the radiation to which the patient is subjected during the examination. There is controversial evidence on the risk of cancer associated with higher radiation levels; however most authorities assume the risk of cancer resulting from X-ray exposure; the risk is higher in younger individuals [19, 20]. The radiation dose to which the patient is subjected in CBCT is 40% lower than in conventional CT and 3 to 7 times higher than in panoramic radiography. The CBCT dose varies substantially depending on the device, FOV, and selected technique factors [21, 22]. Ludlow et al. [22] carried out a study comparing the effective dose of three types of commercially available CBCT equipment, observing that the NewTom 3G emitted the lowest radiation dose, followed by the I-CAT and the CB Mercuray. Pauwels et al. [23] carried out a study to estimate the effective dose absorbed by each organ, using 14 types of CBCT machine (3D Accuitomo 170, Kodak 9000 3D, Galileos Comfort, I-CAT New Generation, Iluma Elite, Kodak 9500, NewTom VG, NewTom VGi, Scanora 3D, Veraviewepocs 3D, Promax 3D, Pax-Uni 3D, Picasso Trio, and SkyView) and different protocols and geometries. These authors conclude that the radiation dose emitted by most machines is in the range of 20 to 100 Sv, confirming that the radiation emitted by CBCT is higher than in the 2D radiographic methods used in dentistry but considerably lower than the doses reported for the common Multislice Computed Tomography protocols. Pauwels et al. [23] observed a big difference in the dose absorbed by the salivary glands, thyroid glands, oral mucous, and the extrathoracic pathways; it is moreover significantly greater with a large FOV than with a medium or small FOV. In the present study the CBCT examination was carried out using a medium FOV, and the patient was not subjected to the highest radiation dose. The same machine can emit different radiation doses for the same examination, depending on operator choices [18, 23]. The Kv and mAs configuration, combined with a large visual field, can also determine a high radiation dose [24]. However it should be noted that when a better quality image is needed, higher exposure factors will be recommended [25].

Dentigerous cysts are usually discovered during routine X-ray examinations or when X-ray is indicated to determine the reason for a failed tooth eruption [26]. Panoramic radiography is a low-cost imaging method widely used in routine dentistry; however it produces a two-dimensional image which only allows two dimensions of the lesion to be observed and does not show its relation to adjacent anatomical structures [27, 28]. Furthermore, because of the superposition of a large tissue volume, extraoral X-ray images often do not provide reliable information on the internal structure of the lesion. Observation of the third dimension, that is, the buccolingual extension of the lesion, requires additional X-rays taken at 90 degrees to the original view [28]. Correct identification of lesions is often impossible in two-dimensional viewing of the image, which may lead to incorrect selection of treatment plan and finally incorrect performance by the dentist. CBCT offers the advantage of a multiplane image (axial, coronal, and sagittal planes) which gives important information on the presence and extension of bone reabsorption, sclerosis of neighbouring bone, cortical expansion, and internal or external calcifications, as well as showing the proximity to other important anatomical structures [28]. Three-dimensional radiographic techniques are of inestimable assistance, not only in differential X-ray diagnosis but also in determining the displacement space of the mandibular canal [29]. We agree with Huumonen et al. [30] and Cohenca et al. [31] when they say that the CBCT image allows in-depth observation of a determined region, eliminating overlapping and so avoiding one of the main limitations of X-rays, the lack of depth. CBCT also offers advantages over Multislice Computed Tomography, including lower radiation emissions, lower cost, and greater accuracy in detecting thinning and/or perforation of the buccal plate and tooth displacement [32]. However it should be noted that CBCT presents the disadvantage of not being indicated for soft tissue analysis. Magnetic Resonance Imaging (MRI) is a noninvasive imaging technique which offers excellent tissue contrast [33] and is becoming increasingly important in oral and maxillofacial surgery. MRI assists in lesion diagnosis because it can reliably differentiate between odontogenic cysts, keratocystic odontogenic tumors, and ameloblastoma, while panoramic radiography and computerised tomography (conventional and cone beam) cannot differentiate between these types of lesion [34]. MRI provides useful information on the relations between the lesion and adjacent structures, as well as offering better tissue contrast and the ability to acquire images along arbitrary planes [35]. MRI presents the additional advantage of not subjecting the patient to ionising radiation; however it is an expensive examination due to the very high cost of the equipment, which puts it beyond the reach of private clinics.

Imaging is an essential part of preparation for most surgical procedures [18]. Preoperative examination of odontogenic lesions prevents complications in surgery and functional deterioration after surgery and reduces surgical stress [32, 36]. In surgical planning for a dentigerous cyst, it may be necessary to measure the lesion from different angles [28], requiring more advanced radiological imaging. CBCT offers great accuracy in measuring osseous components, with less than 1% error as compared to the gold standard method [37]. The CBCT images obtained in the case reported here allowed the margins and limits of the lesion and adjacent structures to be examined, showing details of the relationship between the lesion and the mandibular canal and also the radicular reabsorption of the left mandibular second premolar and the left mandibular first molar. After examining the margins of the lesion in the CBCT images we observed that the patient presented a cystic lesion of expressive magnitude in the body of the mandible region, left side, very close to important anatomical structures. This finding allowed careful planning of surgery, which helped to reduce the time taken in the procedure and the patient's recovery.

In general, surgery is recommended for dentigerous cysts because they often block tooth eruption, become large, displace teeth, destroy bone, and invade vital structures; in some cases they may cause fractures [12, 26]. In selecting the treatment method for a dentigerous cyst, the size and location of the cyst, the age of the patient, the dentition involved, and the participation of vital structures must all be considered [38]. Small cysts which present no risk of lesion to important anatomical structures can easily be enucleated and sent for pathological examination, preserving the tooth and the affected region [15, 38]; this is the technique most frequently used by dental surgeons [10, 12, 13]. Enucleation of the cyst with tooth extraction is indicated for teeth such as the 3rd molar, but if the cyst is very extensive or involves teeth which are important aesthetically or for occlusion, tooth extraction is not indicated. Extraction of the impacted tooth is indicated when the tooth is useless or space is required for eruption [38]. Marsupialisation and decompression are indicated when there are tooth displacement and bone loss [39, 40]. The advantage of marsupialisation is that an impacted tooth can enter into eruption more rapidly [41]; however its main disadvantage is that the pathological tissue remains in situ [39, 42], with the possibility of a more aggressive lesion in the residual tissue [42]. Both enucleation and decompression will relieve the pressure on the cyst, allowing the retained tooth to erupt normally if root formation is incomplete; otherwise the teeth are assisted by orthodontic treatment [38]. In our study, due to the size and wide limits of the cyst, as well as the fact that it was very close to important anatomical structures, we opted for decompression and subsequent enucleation.

According to Neville et al. [12], the level of recurrence of dentigerous cysts is low (3.7%); however patients are recommended to have periodical clinical and imaging monitoring, so we duly informed our patient of the importance of long-term check-ups.

In conclusion we may say that CBCT is a very useful complementary tool for diagnosis and surgery planning in cases of dentigerous cyst. Three-dimensional viewing of the structures using this imaging method offers greater accuracy in planning surgical treatment, thus allowing more effective results to be achieved.

## References

[1] Regezi J. A., Sciubba J. J., Jordan R. C. K. (2003). *Oral Pathology: Clinical Pathologic Correlations*.

[2] Benn A., Altini M. (1996). Dentigerous cysts of inflammatory origin: a clinicopathologic study. *Oral Surgery, Oral Medicine, Oral Pathology, Oral Radiology, and Endodontics*.

[3] Lin L. M., Ricucci D., Lin J., Rosenberg P. A. (2009). Nonsurgical root canal therapy of large cyst-like inflammatory periapical lesions and inflammatory apical cysts. *Journal of Endodontics*.

[4] Lima R. K. P., Faria-Júnior N. B., Guerreiro-Tanomaru J. M., Tanomaru-Filho M. (2010). Diagnosis and planning in apical surgery: use of cone-beam tomography. *South Brazilian Dentistry Journal*.

[5] Yilmaz U. N., Yaman F., Atilgan S. S. (2012). MR *T*_1_ and *T*_2_ relaxations in cysts and abscesses measured by 1.5 T MRI. *Dentomaxillofacial Radiology*.

[6] Pinto A. S., Costa A. L., Galvão N. d., Ferreira T. L., Lopes S. L. (2016). Value of magnetic resonance imaging for diagnosis of dentigerous cyst. *Case Reports in Dentistry*.

[7] Scarfe W. C., Levin M. D., Gane D., Farman A. G. (2009). Use of cone beam computed tomography in endodontics. *International Journal of Dentistry*.

[8] Avelar R. L., Antunes A. A., Carvalho R. W. F., Bezerra P. G. C. F., Oliveira Neto P. J., Andrade E. S. S. (2009). Odontogenic cysts: a clinicopathological study of 507 cases. *Journal of Oral Science*.

[9] Deepthi P. V., Beena V. T., Padmakumar S. K., Rajeev R., Sivakumar R. (2016). A study of 1177 odontogenic lesions in a South Kerala population. *Journal of Oral and Maxillofacial Pathology*.

[10] Ustuner E., Fitoz S., Atasoy C., Erden I., Akyar S. (2003). Bilateral maxillary dentigerous cysts: a case report. *Oral Surgery, Oral Medicine, Oral Pathology, Oral Radiology, and Endodontics*.

[11] Freitas D. Q., Tempest L. M., Sicoli E., Lopes-Neto F. C. (2006). Bilateral dentigerous cysts: review of the literature and report of an unusual case. *Dentomaxillofacial Radiology*.

[12] Neville W., Damm D. D., Allen C. M., Bouquot J. E. (2008). *Patologia Oral & Maxilofacial*.

[13] Shafer W. G., Hine M. K., Levy B. M. (1987). *Tratado de Patologia Bucal*.

[14] Hyomoto M., Kawakami M., Inoue M., Kirita T. (2003). Clinical conditions for eruption of maxillary canines and mandibular premolars associated with dentigerous cysts. *American Journal of Orthodontics and Dentofacial Orthopedics*.

[15] de Carvalho I. K. F., Luna A. H. B. (2016). Spontaneous eruption of premolar associated with a dentigerous cyst. *Case Reports in Dentistry*.

[16] Tsiklakis K., Syriopoulos K., Stamatakis H. C. (2004). Radiographic examination of the temporomandibular joint using cone beam computed tomography. *Dentomaxillofacial Radiology*.

[17] Cha J.-Y., Mah J., Sinclair P. (2007). Incidental findings in the maxillofacial area with 3-dimensional cone-beam imaging. *American Journal of Orthodontics and Dentofacial Orthopedics*.

[18] Horner K. (2013). Cone-beam computed tomography for oral surgical applications: where is the evidence?. *Oral Surgery*.

[19] Wall B. F., Haylock R., Jansen J. T. M., Hillier M. C., Hart D., Shrimpton P. C. (2011). *HPA-CRCE-028—Radiation Risks from Medical X-Ray Examinations as a Function of the Age and Sex of the Patient*.

[20] Mobbs S. F., Muirhead C. R., Harrison J. D. (2010). *HPA-RPD-066—Risks from Ionising Radiation*.

[21] Frederiksen N. L., Benson B. W., Sokolowski T. W. (1994). Effective dose and risk assessment from film tomography used for dental implant diagnostics. *Dentomaxillofacial Radiology*.

[22] Ludlow J. B., Davies-Ludlow L. E., Brooks S. L., Howerton W. B. (2006). Dosimetry of 3 CBCT devices for oral and maxillofacial radiology: CB Mercuray, NewTom 3G and i-CAT. *Dentomaxillofacial Radiology*.

[23] Pauwels R., Beinsberger J., Collaert B. (2012). Effective dose range for dental cone beam computed tomography scanners. *European Journal of Radiology*.

[24] Loubele M., Jacobs R., Maes F. (2006). Radiation dose vs. image quality for low-dose CT protocols of the head for maxillofacial surgery and oral implant planning. *Radiation Protection Dosimetry*.

[25] Lofthag-Hansen S., Thilander-Klang A., Gröndahl K. (2011). Evaluation of subjective image quality in relation to diagnostic task for cone beam computed tomography with different fields of view. *European Journal of Radiology*.

[26] Regezi J. A., Sciubba J. J., Pogrel M. A. (2000). *Atlas of Oral and Maxillofacial Pathology*.

[27] Junqueira R. B., Verner F. S., Vilela E. M., Devito K. L., Chaves M. G. A. M., Carmo A. M. R. (2011). Tomografia computadorizada de feixe cônico como instrumento complementar de diagnóstico e planejamento cirúrgico de cisto radicular: relato de um caso clínico. *Revista de Odontologia da UNESP*.

[28] Ahmad M., Jenny J., Downie M. (2012). Application of cone beam computed tomography in oral and maxillofacial surgery. *Australian Dental Journal*.

[29] McCrea S. (2009). Adjacent dentigerous cysts with the ectopic displacement of a third mandibular molar and supernumerary (forth) molar: a rare occurrence. *Oral Surgery, Oral Medicine, Oral Pathology, Oral Radiology and Endodontology*.

[30] Huumonen S., Kvist T., Gröndahl K., Molander A. (2006). Diagnostic value of computed tomography in re-treatment of root fillings in maxillary molars. *International Endodontic Journal*.

[31] Cohenca N., Simon J. H., Roges R., Morag Y., Malfaz J. M. (2007). Clinical indications for digital imaging in dento-alveolar trauma. Part 1: traumatic injuries. *Dental Traumatology*.

[32] Shweel M., Amer M. I., El-Shamanhory A. F. (2013). A comparative study of cone-beam CT and multidetector CT in the preoperative assessment of odontogenic cysts and tumors. *Egyptian Journal of Radiology and Nuclear Medicine*.

[33] Lenz M., Greess H., Baum U., Dobritz M., Kersting-Sommerhoff B. (2000). Oropharynx, oral cavity, floor of the mouth: CT and MRI. *European Journal of Radiology*.

[34] Minami M., Kaneda T., Ozawa K. (1996). Cystic lesions of the maxillomandibular region: MR imaging distinction of odontogenic keratocysts and ameloblastomas from other cysts. *American Journal of Roentgenology*.

[35] Hisatomi M., Asaumi J.-I., Konouchi H., Shigehara H., Yanagi Y., Kishi K. (2003). MR imaging of epithelial cysts of the oral and maxillofacial region. *European Journal of Radiology*.

[36] Nakagawa Y., Kobayashi K., Ishii H. (2002). Preoperative application of limited cone beam computerized tomography as an assessment tool before minor oral surgery. *International Journal of Oral and Maxillofacial Surgery*.

[37] Ludlow J. B., Laster W. S., See M., Bailey L. J., Hershey H. G. (2007). Accuracy of measurements of mandibular anatomy in cone beam computed tomography images. *Oral Surgery, Oral Medicine, Oral Pathology, Oral Radiology and Endodontology*.

[38] Motamedi M. H. K., Talesh K. T. (2005). Management of extensive dentigerous cysts. *British Dental Journal*.

[39] Takagi S., Koyama S. (1998). Guided eruption of an impacted second premolar associated with a dentigerous cyst in the maxillary sinus of a 6-year-old child. *Journal of Oral and Maxillofacial Surgery*.

[40] Wong M. (1991). Surgical fenestration of large periapical lesions. *Journal of Endodontics*.

[41] Miyawaki S., Hyomoto M., Tsubouchi J., Kirita T., Sugimura M. (1999). Eruption speed and rate of angulation change of a cyst-associated mandibular second premolar after marsupialization of a dentigerous cyst. *American Journal of Orthodontics & Dentofacial Orthopedics*.

[42] Hupp J. R., Tucker M. R., Ellis E. (2014). *Contemporary Oral and Maxillofacial Surgery*.

